# Dynamics of a Tularemia Outbreak in a Closely Monitored Free-Roaming Population of Wild House Mice

**DOI:** 10.1371/journal.pone.0141103

**Published:** 2015-11-04

**Authors:** Akos Dobay, Paola Pilo, Anna K. Lindholm, Francesco Origgi, Homayoun C. Bagheri, Barbara König

**Affiliations:** 1 Institute of Evolutionary Biology and Environmental Studies, University of Zurich, Zurich, Switzerland; 2 Institute for Veterinary Bacteriology, Vetsuisse Faculty, University of Bern, Bern, Switzerland; 3 Centre for Fish and Wildlife Health (FIWI), Vetsuisse Faculty, Bern, Switzerland; CEFE, FRANCE

## Abstract

Infectious disease outbreaks can be devastating because of their sudden occurrence, as well as the complexity of monitoring and controlling them. Outbreaks in wildlife are even more challenging to observe and describe, especially when small animals or secretive species are involved. Modeling such infectious disease events is relevant to investigating their dynamics and is critical for decision makers to accomplish outbreak management. Tularemia, caused by the bacterium *Francisella tularensis*, is a potentially lethal zoonosis. Of the few animal outbreaks that have been reported in the literature, only those affecting zoo animals have been closely monitored. Here, we report the first estimation of the basic reproduction number *R*_0_ of an outbreak in wildlife caused by *F*. *tularensis* using quantitative modeling based on a susceptible-infected-recovered framework. We applied that model to data collected during an extensive investigation of an outbreak of tularemia caused by *F*. *tularensis* subsp. *holarctica* (also designated as type B) in a closely monitored, free-roaming house mouse (*Mus musculus domesticus*) population in Switzerland. Based on our model and assumptions, the best estimated basic reproduction number *R*_0_ of the current outbreak is 1.33. Our results suggest that tularemia can cause severe outbreaks in small rodents. We also concluded that the outbreak self-exhausted in approximately three months without administrating antibiotics.

## Introduction

Tularemia is a zoonotic disease caused by the bacterium *Francisella tularensis*. This microorganism is widespread in the Northern Hemisphere [1]. *F*. *tularensis* is able to infect an exceptionally large number of animal species, ranging from unicellular organisms to mammals [1, 2]. Tularemia is associated with debilitating clinical manifestations and is a potentially lethal disease in humans. Cases mainly occur as sporadic events in humans and in animals, but outbreaks can arise when the source of infection is widely spread and / or many people or animals are exposed to it [3–6]. However, outbreaks are rare and very difficult to monitor or trace, since the most commonly affected species are wild rodents and lagomorphs [1, 7, 8]. Few reports exist investigating outbreaks of tularemia in semi-free-living and indoor / outdoor-housed groups of primates [9–11]. In humans, person-to-person transmission of *F*. *tularensis* has not been reported, and people can become infected with this microorganism by all five classical routes of transmission: ingestion, inhalation, direct contact with a contaminated source, animal bites, and by arthropod intermediates [12]. In animals, experimental infections showed that all those ways of transmission are effective, but the question of transmission from individual to individual is still open, and transmission via cannibalism or excrement cannot be ruled out at the moment [13]. Irrespective of the species and transmission route involved, understanding the pathogenicity of this microorganism constitutes an important aspect for controlling the disease [14–16]. This can be achieved in the laboratory by infecting animals to study the interaction of the bacterium in host cells or with the immune system of the whole animal. Ultimately, these studies should help develop medical countermeasures against the disease [17–19].

Several ecological, genetic or phylogenetic studies have investigated the statistical distribution of tularemia cases [20, 21]. Using a regression model, Rydén *et al*. collected environmental data to correlate the presence of tularemia disease in humans with the prevalence of mosquitos in Sweden [22]. Modeling of infectious diseases in humans, animals and plants is relevant to understanding and forecasting outbreak dynamics. Together with statistical data, this approach also provides information important for decision makers who establish sanitary policies for outbreak management. Gee *et al*. developed a susceptible-infected-recovered (SIR) framework to model the impact of a live vaccine against tularemia [23]. Attie *et al*. used *in silico* modeling (agent-based) and existing experimental data to investigate the outcome of an infection in both human and mouse lung and liver with *F*. *tularensis* [24] (for a similar study see also [16]).

Here we report the modeling of a natural outbreak of tularemia in the field, using a basic SIR framework with births and deaths [25]. The outbreak occurred in 2012 in a closely monitored, free-roaming wild house mouse (*Mus musculus domesticus*) population near Zurich, Switzerland. This population has been intensively investigated in studies of behavior and ecology since 2002 [26, 27]. Births and deaths are regularly monitored by inspection of all nest boxes and surfaces, and carcasses are removed when found. The outbreak was initially suspected because of the sudden increase of deaths among the mice, which were then sent for *postmortem* examination. Collected data were used to estimate the basic reproduction number *R*_0_ of tularemia for the present outbreak. This information is relevant to better understand the dynamics of natural tularemia outbreaks. Moreover, a heuristic value to determine an appropriate sampling size in further epidemiological studies investigating tularemia in wildlife was derived from the number of animals confirmed to be affected by tularemia in the whole population [28]. This value is based on the observation of how many mice were necessary to detect infected individuals and assess the end of the epidemic, but is likely to vary depending on the population size and the force of the infection [29].

## Materials and Methods

### Ethics Statement

The permit for the field study on house mice was issued by the Veterinary Office Zurich, Switzerland (Kantonales Veterinäramt Zürich, no. 51/2010).

### Study Population and diagnostic methods

In 2002, 12 house mice trapped in the local Illnau area were introduced to a 72 m^2^ agricultural building that allows mice to enter and exit, but is inaccessible to predators. The population has grown ever since [26]. The concrete floor of the building is covered with standard mouse cage bedding, and tubes, bricks and sticks provide hiding places. Nest boxes and straw are provided for breeding. Water bottles and food, a mixture of oats and commercial rodent food, are provided *ad libitum*. Since 2002, the population has been intensively monitored, with 2–3 visits per week depending on season. Checks of nest box contents are regularly made and the entire population is captured every two months. All dead mice found are recorded and immediately removed and frozen. The research has focused on breeding strategies of females [27].

In early June 2012, the number of dead mice suddenly increased, and most of the dead mice had no visible injuries. Wounds are usually symptomatic that intraspecific aggression caused the death of an individual. Sixty-nine carcasses collected between May 2012 and June 2013 were tested for the presence of *F*. *tularensis* by culture in a selective medium and by direct PCR, targeting the *fopA* gene as previously described [30]. Moreover, isolates were identified at the subspecies level by amplifying the RD1 locus by PCR [30].

### Modeling Toolbox

The basic susceptible-infected-recovered (SIR) model assumes the existence of individual states [25, 31]. Susceptible *S* and infected *I* individuals are in contact within a homogeneous population. The force of infection is the rate at which susceptible individuals become infected. In our case, the force of infection is a combination of the probability of encounter *per capita* between susceptible and infected individuals, and the probability to transmit the infection. Infected individuals recover or die after a period. These are called recovered or removed individuals *R*.

An outbreak can be characterized by the value *R*_0_, the basic reproduction number [25, 32, 33] (see below). Information about the force of infection, the recovery rate and the population size of susceptible individuals can be combined to calculate this number. The value *R*_0_ indicates the number of secondary infections produced by one primary infection in a population of susceptible individuals. This value provides critical information to policy makers concerning the potential spread of the infection within a population and its likely rate of transmission. If *R*_0_ > 1 the number of infected will increase, while if *R*_0_ < 1 the disease will die out. Therefore the value *R*_0_ can be used by policy makers to know whether the number of infected will rise at first in a given population size. The value *R*_0_ in a partially immune population is also of interest for policy makers if available.

Given a *per capita* force of infection *k*_1_, a recovery rate *k*_2_, a time-dependent birth rate *per capita k*_3_(*t*), a natural death rate *per capita k*_4_, and a time-dependent virulence rate *per capita k*_5_(*t*), the model is expressed as

$$dS/dt=-k_{1}SI+k_{3}\left(t\right)-k_{4}S$$ (1)

$$dI/dt=k_{1}SI-k_{2}I-k_{4}I-k_{5}\left(t\right)$$ (2)

$$dR/dt=k_{2}I-k_{4}R$$ (3)

The time-dependent parameters *k*_3_(*t*) and *k*_5_(*t*) are step or piecewise functions of the form

$$k_{3}\left(t\right)=\{\begin{array}{ll} \;0.014 & \;0<t<30\\ \;0.015 & \;30<t<60\\ 0.007 & 60<t<120 \end{array}$$ (4)

and

$$k_{5}\left(t\right)=\{\begin{array}{c} 0\;0<t<30\\ 0.001\;30<t<90\\ 0.0002\;90<t<120 \end{array}$$ (5)

We estimated *k*_5_(*t*) using our record of tularemia positive dead mice. For this model, the basic reproduction number is given by

$$R_{0}=k_{1}S_{0}/\left(k_{2}+k_{4}\right)$$ (6)

The value *S*_0_ represents the entire population of susceptible individuals at the time *t* = 0. It is worth noting that information collected from dead or recovered individuals cannot be used to trace back the mode of transmission of the pathogen. An estimate of the force of infection *k*_1_, and subsequently the total number of infected individuals *I*, can be obtained using a fitting procedure of the total number of removed individuals following an infection (see below). To remain consistent with our notations, we will write *I*_0_ to represent the number of infected individuals at *t* = 0. To avoid any ambiguity with respect to *R*_0_, we will write *R*_*t* = 0_ to indicate the number of recovered or removed individuals a *t* = 0.

Tularemia can be directly and indirectly transmitted. A simple way to quantify non-contact transmissions of a pathogen in the SIR model is to introduce an additional rate *k*_6_ at which susceptible individuals become indirectly infected. Accordingly, Eqs 1 and 2 can be changed as follows

$$dS/dt=-k_{1}SI+k_{3}\left(t\right)-k_{4}S-k_{6}S$$ (7)

$$dI/dt=k_{1}SI-k_{2}I-k_{4}I-k_{5}\left(t\right)+k_{6}S$$ (8)

The total number of dead mice recorded in June, July and August 2012 was 148 (see Table 1). Given that not all the carcasses of dead mice could be investigated for tularemia because of their advanced autolytic stage, we had to estimate the fraction that was likely caused by the disease. This fraction (or estimate) for June, July and August was obtained by counting the total number of positive individuals divided by the total number of tested mice (see S1 Table). We found an average relative frequency of 58.3% positives among adults and 75.0% among subadults. However, for modeling the disease we did this calculation for each month separately, and applied the estimators to account for the actual number of dead mice recorded in June, July and August (see S2 Table for more details). In September this fraction was zero.

**Table 1:** Time progression of the number of deaths during the outbreak.

Month	Number of dead adults	Number of dead subadults	Number of dead adults tested positive (out of)	Number of dead subadults tested positive (out of)
**June**	47	16	9 (9) 100%	2 (2) 100%
**July**	38	15	10 (16) 62.5%	1 (1) 100%
**August**	25	7	2 (10) 20%	0 (1) 0%

Progression of the tularemia outbreak based on the total number of dead adults and subadults found in the study population between June and August 2012, and the corresponding number of adults and subadults tested positive with tularemia among all tested animals (number given in brackets). The value in percent corresponds to the estimator. Note that no positive-tested mouse was found prior to June and after August (see S1 and S2 Tables for more details).

The solutions were obtained using numerical routines in Matlab version 2014b. We used a Nelder-Mead optimization algorithm to fit our experimental data (Matlab functions fminsearch and fminunc). The set of differential equations were solved using the Matlab solver ode45 for non-stiff differential equations. The optimization procedure used a simple Euclidian distance function *f*_*R*_ (objective function) for the number of removed individuals as a measure of the quality of the fit.

### Model parameter and parameter optimization

For the modeling, a total of 40 mice, including adults and subadults, were used. Tularemia was diagnosed in 24 house mice (21 adults and three subadults) by PCR between June and August 2012 (see Table 1). Eight isolates recovered from eight distinct animals out of the 24 PCR positive mice could be cultivated. After the suspicion and confirmation of the first tularemia cases, all collected dead mice were sent to the Institute for Veterinary Bacteriology in Bern, Switzerland, where carcasses were necropsied and tested for *F*. *tularensis*. Identification of the isolates by culture and direct PCR confirmed that the disease was caused by *F*. *tularensis* subsp. *holarctica* (type B) [30]. In addition, 14 mice found dead in September 2012 were tested for *F*. *tularensis* by PCR. None of them were diagnosed with tularemia.

We then plotted the estimated cumulative sum of all positive dead mice between June and August, which corresponds to a total of 106.8 (see S2 Table for more details). We followed two different fitting strategies while solving the differential equations of the SIR model (Eqs 1–3). In one case, we used a laboratory determined average removal rate of *k*_2_ = 0.12, obtained for mice infected with two strains of *F*. *tularensis* subsp. *holarctica* isolated in 1999 and 2000 in Kentucky and Michigan (USA), respectively [29]. The value *k*_2_ = 0.12 corresponds to a 50% likelihood of survival (on average half of the infected mice were dead after 200 hours). This value was obtained by directly injecting the pathogen in higher concentration than the usual dosage received from natural exposure in the wild. The mortality rates obtained in [29] might therefore constitute an overestimation for *k*_2_. Nevertheless, we optimized the fitting procedure with the constraint of keeping *k*_2_ constant.

The number of births was taken from our nest monitoring between May and August 2012. The number of births corresponds to the number of pups found dead or alive in litters that were found for the first time. May and June coincided with a higher reproductive activity, accounting for 153 and 165 births respectively. Following the onset of the outbreak, this number was halved (81 in July and 80 in August). We then divided the daily rate by *S*_0_ to obtain a *per capita* value of 0.014 in May, 0.015 in June and 0.007 in July and August. We used these estimators to create a time-dependent birth rate for our model (see Eq 4).

The number of deaths from other causes than tularemia was estimated from our monthly population capture in the preceding year, 2011. As the population was increasing in size since 2002, this is the most reliable estimate we can obtain for a density-dependent death rate between May and August in the absence of tularemia. For our estimate we pooled the number of deaths from May to August. In our study population natural death occurs during the whole year and is only marginally affected by seasons, which is clearly not the case for births. However, we excluded pups from our death rate and considered only adults and subadults. Mortality rate among pups is significantly higher, most likely because of infanticide [34]. Adult and subadult mortality comprised eight in May, 18 in June, 30 in July and 21 in August 2011. We converted these values into a daily rate *per capita* of 0.002.

To achieve the fit, while keeping *k*_2_ = 0.12 constant (constrained), we set the initial number of infected to *I*_0_ = 1 (at time *t* = 0) for the number of infected mice. The start of the epidemic was estimated to be at the beginning of May 2012, even if no mice testing positive were found during this month. Setting the time *t* = 0 to the beginning of June, as initially suggested by our data, resulted in a poor fit, unless we increased the initial number of infected to *I*_0_ = 18 (constrained) and *I*_0_ = 3 (unconstrained). To estimate the beginning of the epidemic we explored several time points in May until Matlab was able to fit the data with *I*_0_ = 1 (see Results and Discussion for more details).

**Fig 1:**
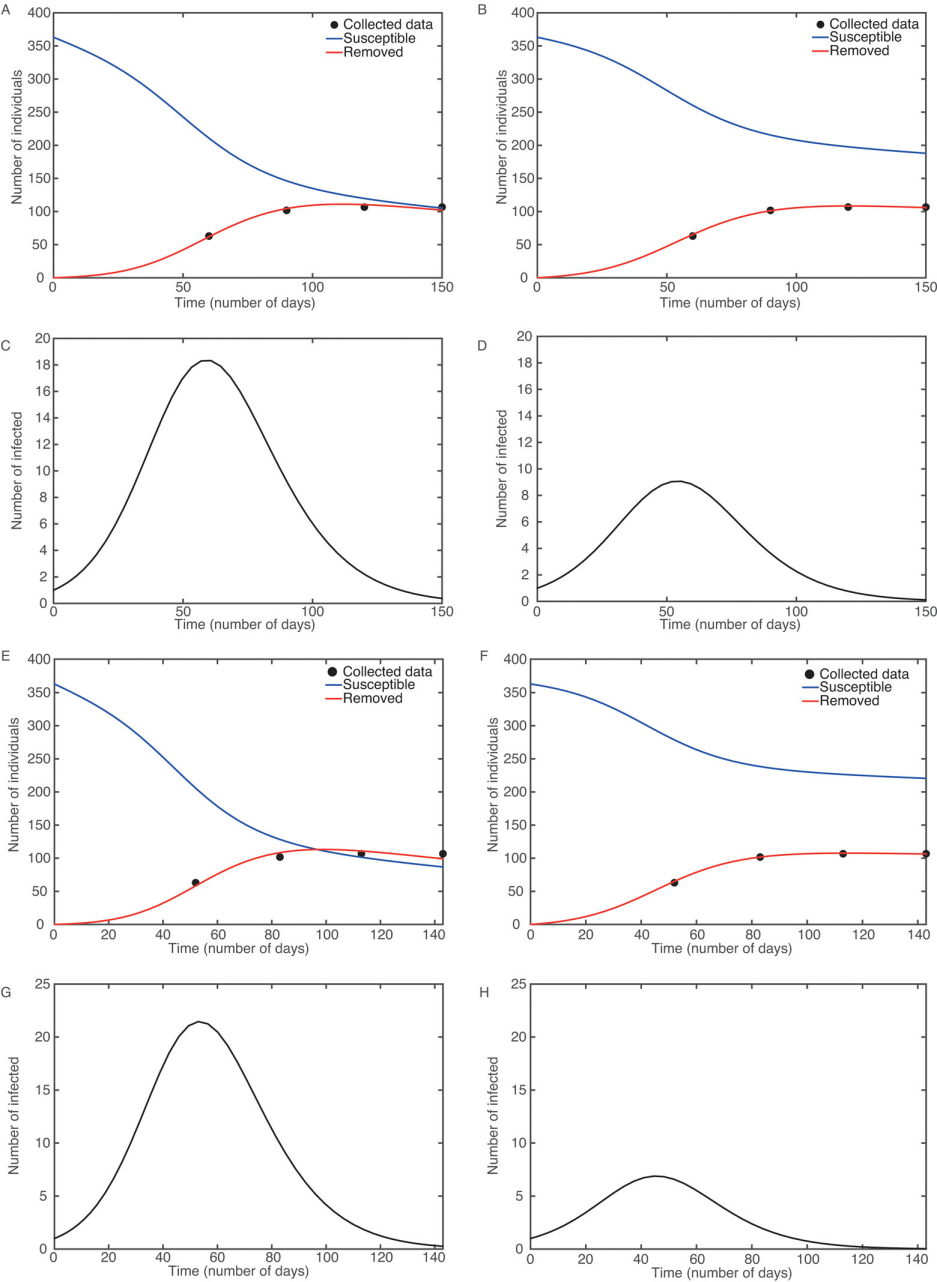
Numerical analysis of the SIR model using Eqs 1–3. Best fit curve with estimated start on May 1: constrained (*k*_2_ = 0.12) in (A) and (C); unconstrained in (B) and (D). Best fit curve with estimated start on May 7: constrained in (E) and (G); unconstrained in (F) and (H).

In the case with *k*_2_ = 0.12, the best constrained fit curve was obtained for *S*_0_ = 364, *I*_0_ = 1, *R*_*t* = 0_ = 0, *k*_1_ = 0.0006, *k*_4_ = 0.004, *k*_6_ = 0 and *f*_*R*_ = 46.89. The fitting procedure resulted in a value of *R*_0_ = 1.70 (Fig 1A and 1C). In the second case, we did not impose any constraint on the recovery rate to explore a different scenario. Fig 1B and 1D show the best unconstrained fit curve for *S*_0_ = 364, *I*_0_ = 1, *R*_*t* = 0_ = 0, *k*_1_ = 0.0008, *k*_2_ = 0.21, *k*_4_ = 0.002, *k*_6_ = 0 and *f*_*R*_ = 4.87. The second fitting procedure resulted in a lower value of *R*_0_ = 1.33. Note that during this last fitting procedure we did not use a constant value *k*_4_, but left it free, which was not the case for *k*_3_(*t*) and *k*_5_(*t*) (see Eqs 4 and 5).

Finally, we explored the possibility of having a single quasi-static source of infection where susceptible mice are exposed and become infected at a given rate *k*_6_, while excluding transmission by body contact (Eqs 7 and 8 with *k*_1_ = 0). Unfortunately, the design of our model did not allow a proper fitting of the data without having unrealistic values for the remaining parameters. This is also explained by the fact that having exclusively *k*_6_ in Eqs 7 and 8, together with *k*_1_ = 0, led to a model that is independent of the number of infected individuals.

## Results and Discussion

This study used information on a natural outbreak of tularemia in free-roaming mice to model the dynamics of the disease. It is a rather unique investigation since monitoring tularemia in wildlife, where affected species are often secretive and rapidly scavenged by predators, is very difficult. Moreover, experimental infections are usually performed with inbred animals that might limit the variation in infection rates observed in the field. Furthermore, captive laboratory conditions do not reproduce natural conditions of transmission and ecological factors involved in a “natural” outbreak. Here, in contrast, we used observations from a relatively natural situation. Several points crucial for further epidemiological studies in wildlife could be observed.

First, the outbreak self-exhausted in approximately three months without any antibiotic treatment. The potential duration of an outbreak is a crucial parameter for managing and taking protection measures for humans and animals during an outbreak of tularemia in wildlife. Table 1 shows the time evolution of the outbreak from June to August. In September no positive-tested mouse was identified out of 14 carcasses found during the same month.

Finding the start of the epidemic also provides valuable information. We therefore allowed the model to go back as far as needed so that *I*_0_ = 1. Setting the start of the epidemic in the middle of May (15^th^) was only possible when fitting the data with *I*_0_ = 8 (best fit). When getting closer to the beginning of May we were able to fit the data while reducing *I*_0_. The earliest time point with *I*_0_ = 1 was the 7^th^ of May according to our calculations and the combination of initial parameters in our model (*k*_1_ = 0.0003, *k*_2_ = 0.12, *k*_4_ = 0.001, *k*_6_ = 0). The final values after fitting the data were *k*_1_ = 0.001, *k*_2_ = 0.29, *k*_4_ = 0.0008, *k*_6_ = 0, *f*_*R*_ = 1.19, and *R*_0_ = 1.24 (see Fig 1F and 1H). With the constrained fit we estimated *R*_0_ = 1.81 (see Fig 1E and 1G). We then pushed this procedure to reach the beginning of May and obtained a slightly poorer fit (*f*_*R*_ = 4.87), but a better match with our usual death rate (*k*_4_ = 0.002) and the laboratory determined average removal rate (*k*_2_ = 0.21). The basic reproduction number was also slightly higher with *R*_0_ = 1.33 (see Materials and Methods and Fig 1A to 1D). It is not straightforward which one of these two sets of solutions should be retained as the final one since both of them provide valuable insights into the outbreak.

Second, we estimated the minimal number of affected animals during a natural outbreak. This number is important to better evaluate the minimal number of animals to be sampled in future studies with similar population size and similar force of infection if the latter information is already available. [28]. Our sampling suggested that a minimum of 10 animals was necessary in order to detect traces of tularemia. It follows from the simple observation that if the number of tested mice was less than 10 in August we might have missed positive cases. This number is based on our case study and most likely cannot be generalized. Nevertheless it is important to provide such information in the absence of robust sampling design (see for instance [35, 36]).

Third, we had to introduce two approximations to estimate *R*_0_ in our study population of house mice infected by *F*. *tularensis* subsp. *holarctica*. First, we extrapolated the total number of mice that died from the disease by the fraction of those that could have been found and tested positive. Second, we used different starting numbers *I*_0_ of infected mice to initiate the model based on Eqs 1–3. As discussed above this value was not known and we had to treat it as a variable in our model. This indicated that June was not the start of the outbreak, but rather May, and, at the time the outbreak became noticeable, several mice were already infected. According to our records, this might be the case as the number of deaths did not increase significantly in July and was decreasing in August (see Table 1). If we do not use the value of *k*_2_ from [29], our modeling result points to an outbreak with *R*_0_ = 1.33 (Fig 1B and 1D). This result also leads to a 2-fold increase in the removal rate compared to the one suggested in [29]; a value close to 120 hours (5 days) instead of the 200 for a survival rate of 50% [29]. It is important to note that the removal rate in the SIR model is a combination of several factors and only indicates how long an individual remains infectious. Hence, the value of *k*_2_ cannot be directly correlated with the virulence of *F*. *tularensis* subsp. *holarctica*. To learn more about the virulence of the strain, an experimental approach would be required.

Even if we cannot directly learn about the virulence, we corrected at least for this limitation by separating usual death (death during years without tularemia) from death due to tularemia. To do so, we introduced an additional rate *k*_5_(*t*) next to the rate of usual death *k*_4_ (Eqs 2 and 8). To estimate the death rate due to the infection we used the number of dead animal tested positive (Table 1). This correction was non-negligible when optimizing the model and estimating the basic reproduction number.

Adding an infection rate *k*_6_ to account for transmissions without direct contact with another infected individual did not provide information about the possibility of having both contact and non-contact infections. To test the extreme case where transmissions would only result from a vector instead of another infected individual, we set *k*_1_ to zero. In such a case, the fitting algorithm could not find a realistic solution. Even if the variation in the number of susceptible animals depends on the population size (*k*_6_ *S*), it is unlikely to simulate the dynamic we observe in our dataset without the interaction term between susceptible and infected individuals (*k*_1_ *SI*). When the interaction term is restored the optimization algorithm will distribute the optimal solution between *k*_4_ and *k*_6_, which is a limitation in our model to explore mixed scenarios. To investigate a mixed scenario, a model with a more complex structure would have been required. Another aspect that might preclude a reliable estimate of *R*_0_ is the effect of cannibalism on the spread of the disease. In that case, we cannot talk about recovery, since only carcasses that have been physically removed from the study population are no longer infectious. Hence the rate of removal corresponds to the rate at which dead bodies were removed. If dead bodies were not removed regularly during every monitoring session, the size of the outbreak could have been larger [30]. In addition, another possibility is that the rate of transmission from the environment is not constant through time. This is likely if necrophagia is an important driver: the more dead mice are present, the more likely necrophagia events would take place. As such, environmental transmission would show some level of temporal trend. However the same limitation in our model as mentioned above will also apply here when searching for temporal trend. One option would be to correlate *k*_6_ with *k*_5_(*t*). We tested the idea, but we were unable to fit the data after choosing several combinations of starting parameters.

Fourth, our modeling approach has its limits, especially for wildlife outbreaks. We made extensive use of modeling to describe an outbreak of tularemia in the wild. We tried to demonstrate that, in the absence of data on the size of the epidemic, formulating an epidemiology model may provide a useful approach to fill the gap. However, the complex social interactions of mice are unlikely to be captured by a rather simple SIR model [26], even if SIR models allow additional refinements such as including structured populations or spatial distributions in case of stochastic models [25]. Environmental factors and potential vectors of the disease also have complex dynamics, which are difficult to incorporate in our approach without dramatically increasing the level of complexity of the equations. By entering the study population to collect data we also inevitably interfere with the course of the outbreak. Another aspect that we omitted in our model is the effect of migration. Emigration from the population accounts for a substantial fraction of the subadult population due to competition for resources. Immigration is also possible during the whole year. Thus, the outbreak could have presumably started by a mouse coming from outside. Some of these aspects can better be addressed using computer simulations such as for instance agent-based models.

Finally, the data did not provide any evidence as to the location of the reservoir and the source of the pathogen [30]. The outbreak appeared in June 2012 and did not show any recrudescence until the present (October 2015), although animals were not treated with antimicrobial agents. This shows how unpredictable outbreaks may be in the wild and confirms the necessity to investigate the ecology of emerging pathogens in detail to better understand how diseases are sustained in specific populations and persist in the environment.

Epidemics in wildlife populations are often difficult to characterize due to the scarcity of data. Contrary to epidemics in human populations, there is no network of medical caregivers and hospitals that leave large amounts of data on the progression of the disease. Hence, the study of wildlife epidemics requires estimation techniques that rely on scarce or incomplete data. However, the corresponding effort is important due to two principal benefits. First, models are always simplifications, and hence an iterative interaction with observational data is essential to make such models more accurate. Second, as the history of the plague has notoriously illustrated, rodent-based zoonoses are often not isolated from human populations, and can serve as a reservoir, which can subsequently initiate a human epidemic [37]. Consequently, modeling and parameterizing rodent-based epidemics is essential for understanding their nature, spread and control.

## Conclusion

The data collected in this study allowed us to elaborate different potential scenarios concerning the dynamics of a natural outbreak of *F*. *tularensis* subsp. *holarctica* in a free-ranging population of house mice and to extrapolate a baseline value that can guide future sampling design in epidemiological studies of tularemia in wildlife. The *R*_0_ value ranged from 1.24 to 1.81 in the worst-case scenario according to the type of assumptions we made in our model, the information gathered from literature and the beginning of the outbreak. These are the first estimates for an outbreak of tularemia in a free-roaming population of mammals. We propose the value *R*_0_ of 1.33 as the closest estimate in line with the data collected during the outbreak and the data obtained from our regular population monitoring.

Our data suggest that tularemia can cause severe outbreaks in small rodents such as house mice. At the same time the outbreak self-exhausted in approximately three months, without treating animals with antibiotics. This information will be relevant for further epidemiological investigations of tularemia outbreaks.

## Supporting Information

S1 Table Date of death of each individual mouse tested for tularemia between May and August 2012. List of all mice found dead during the outbreak and investigated for tularemia. The mice used in the modeling are indicated in the third column. Pups were excluded in the modeling. The likelihood of death due to infanticide is very high among pups; when they are found dead, it is not straightforward to establish the cause of death. Note that no positive-tested pups were found during the outbreak. The date in the first column indicates the day when a mouse was found dead in the study population (in Microsoft Excel format).
(XLSX)

S2 Table Number of dead mice between June and August 2012. Time progression of the number of deaths between June and August 2012. The table contains the raw data and the corrected values used for the modeling (in Microsoft Excel format).
(XLSX)
